# Naloxone administration by nonmedical providers- a descriptive study of County sheriff department training

**DOI:** 10.1186/s13011-020-00327-w

**Published:** 2020-11-12

**Authors:** Alan Janssen, Brittany Garove, Virginia LaBond

**Affiliations:** Ascension Genesys Hospital, One Genesys Parkwa, Grand Blanc, Mi 48439 USA

**Keywords:** Non-medical naloxone administration

## Abstract

**The study background:**

In 2015 a county sheriff department in Michigan began a training program for its deputies on administration of naloxone for non-medical providers.

**Methods:**

A descriptive analysis was used to evaluate the effectiveness of the program. Data collected from the Sheriff’s department allowed the study to quantify the incidence of naloxone administration, describe characteristics related to the administration, and report on aggregate outcomes.

**Results:**

Of the reported 184 incidents involving naloxone use the sheriff department had an overall successful administration rate of 94.6% in the cases from 2015 to 2017. It was also noted that the overall number of naloxone administrations showed an upward trend with a greater number of trained deputies.

**Conclusion:**

The outcome of training non-medical first responders in naloxone administration has been shown to be successful with regard to resuscitation of patients with opioid overdose.

## Introduction

There are few communities today naive to the burdensome impact of rising narcotic abuse, since the US Department of Health and Human Services declared it as a nationwide public health threat in October 2016 [1]. The class of narcotics includes prescribed medications such as morphine, methadone, hydrocodone, and oxycodone as well as other illicit opioids. Other illicit opioids include heroin and fentanyl. With a rise in the use of narcotics there is also a subsequent increase in the number of overdoses and deaths. The CDC reported over 47,000 opioid related deaths in the US in 2017 [2]. This amounts to an average of 130 people daily. Compared to 1999, these numbers have escalated six-fold [3].

The growing trend was initially noted in the 1990s, around the time of an upsurge in narcotic prescribing. Despite changes in physician prescribing practices since that time, narcotic use grows with the availability of inexpensive heroin and synthetic opioids [3]. These substances can often have high potency, with illicitly manufactured fentanyl being 50 times that of heroin, and carfentanyl having 10,000 times the potency of morphine [4].

Massive efforts are underway at both the local and federal levels to curtail this expanding crisis. There has been a focus on education of both the public and healthcare professionals as well as providing resources to those currently suffering opioid addiction [3]. Foreign policies have been enacted to help reduce the influx of illicit substances into the US [5]. Also, the medication Naloxone has become widely available and proven effective in treating the acute overdose victims [6].

Naloxone, an opioid antagonist, binds to the mu receptors within the nervous system, blocking and quickly reversing the effects of opioids with limited potential for harmful side effects [6]. This is a critical medication to prevent the well-known dangers of opioid overdose being asphyxiation, cardiac arrest, and death. Time to administration of naloxone is key to prevent irreversible brain death that begins minutes after someone becomes unresponsive and in respiratory arrest. Initially this medication was used only in hospitals by trained physicians and nurses through intravenous administration. However, the challenge to reach overdoses outside the hospital and into people’s homes quickly, necessitated the development of an intranasal administration which has proven to be used successfully [6].

Emergency medical services (EMS) are widely equipped with intranasal naloxone. Pharmacies and programs around the nation have also begun to train lay people to administer the medication. In the same way that lay people improve outcomes by administering CPR in a cardiac arrest, they can provide a similar benefit in narcotic overdoses by shortening the time to the reversal agent [7]. Non-medical police officers are one important target population for training, as they are often the first to arrive at the scene of a crisis.

A county sheriff’s office in Southeastern, Michigan developed a naloxone administration training program for deputies starting in January 2015. This was one of several actions being taken by the county with yearly statistics indicating, like many other places, a growing opioid problem. In 2016 there were 743,969 opioid prescriptions filled in the study county and 165 opiate confirmed deaths [8]. The deputy training has been ongoing since 2015 with continuous successful resuscitation outcomes from nonmedical providers. While the exact total number of deputies employed in the county is unknown, it is known that there are 15 contractual divisions with a range of 4–75 deputies employed per division [9]. The goal is to train all of them on naloxone administration.

The training consists of 45 min of lecture followed by a hands-on practical application session. The lecture topics included signs and symptoms of opioid abuse, physiology of opioid use, recognition of opioid overdose as well as the pharmacology and safe administration of an opioid antagonist [10]. A simulated victim model is then used by the trainees for practice of the simple maneuver, which included patient positioning, placing the syringe tip in the patient’s nostril, and providing a firm press to administer medication. We evaluated characteristics of each incident of naloxone administration that were recorded once the program was initiated. The aim of this study was assess the impact of the training program.

## Materials and methods

The sheriff’s office database with all naloxone administrations by non-medical deputies was accessed to obtain the data collected for this study. It includes a record of all administrations of naloxone since the initiation of the program. Internal Review Board (IRB) approval was obtained on July 2, 2019. We retrospectively collected the known data on the incidents from the first administration on February 8th, 2015 through January 31st, 2018. The data points included the number of naloxone doses administered for each case as well as whether or not the administration of naloxone resulted in a successful resuscitation. This database also included demographic information including gender, age, and race. Information obtained outside of the database includes the number of deputies trained per year between 2015 and 2018.

### Statistical analysis

Using this information, we assessed the characteristics of events in which sheriff deputies administered naloxone. This was a descriptive study to quantify the incidence of naloxone administration, describe characteristics related to the administration, and report on aggregate outcomes.

## Results

There was a total of 184 reported incidents in which the sheriff deputies administered naloxone from 2015 to 2018. The average number of doses administered was one (range 1–3 doses). The average age of the participants to receive naloxone was 35.02 years (range of 6 years to 71 years). The patients were 64.7% male (*n* = 119) and predominantly Caucasian (85.3%) *n* = 157. The largest number of deputies who trained in naloxone administration was in the year 2016 (*n* = 198 [range 62–198]).

Administrations of naloxone were successful in 94.6% of cases (*n* = 174). When following the administrations per year, the most naloxone administrations occurred in 2017 with 38% of administrations (*n* = 71). The most unsuccessful administrations of naloxone by year occurred in 2017 as well at 9.9% (*n* = 7).

Table 1 demonstrates the number of deputies, the number of naloxone administrations, the successful administrations, and the unsuccessful administrations by year.

**Table 1:** Characteristics of Naloxone administration by year

Year	Number of Deputies Trained	Number of Naloxone Doses Administered	Number of Successes	Unsuccessful Administrations
2015	105	20	19 (95%)	1 (5%)
2016	198	48	47 (98%)	1 (2%)
2017	143	71	64 (90%)	7 (10%)
2018	62	45	44 (98%)	1 (2%)
Total	508	184	174 (95%)	10 (5)

**Fig. 1:**
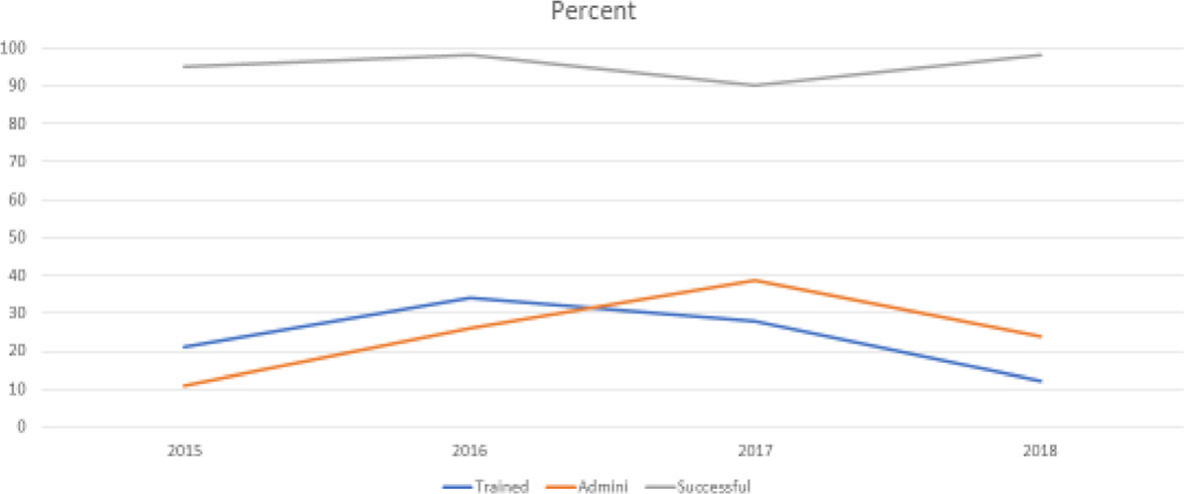
Trained Deputies, Administrations, and Successes as a Percent of the Overall Totals

Figure 1 shows the percentage of overall trained deputies, the percentage of overall naloxone administrations, and the percentage of successful resuscitations by year.

## Discussion

Review of the demographic findings of patients treated with naloxone was largely expected. The patients had an average age of 35, which is consistent with the findings reported by the National Center for Health Statistics [11]. According to the National Institute on Drug Abuse men are more likely to abuse almost all illicit substances, including opioids, as well as require medical attention supporting the predominance of male gender (65%) in this study [3]. The U.S. Census Bureau reports an estimated 75% of the study county population is Caucasian [12] which explains the high percentage of Caucasian patients (85.3%).

We were pleased to see that the administration of naloxone was successful in almost 95% of cases. Although we don’t have details regarding the decision making processes of the deputies, it appears that they have effectively implemented this training program.

Table 1 shows a peak in the number of deputies trained per year in 2016, an 80% increase from the previous year. Interestingly the following year, 2017, had a 50% increase in the number of naloxone administrations. This trend is clearly demonstrated on Fig. 1 with the peak of training appearing in the year prior to the peak in naloxone administrations. Although there are multiple factors that could lead to an increase in successful naloxone administration, we believe the increase in number of trained deputies may be one contributing factor.

Overall, Naloxone administrations increased as the number of trainees increased and remained on an upward increase even as the number trained declined. This is may be due to the fact that each year, fewer trainees need the training and those who were previously trained retained their comfort in administering the naloxone. However, successful outcomes declined during the year of most administrations and then increased again when administrations decreased.

The implementation of the training of lay providers has shown success with regard to naloxone administration by trained deputies. Although this study is unable to assess the overall impact on U.S public health, further funding for more training and studies of such training programs around the nation may demonstrate more successful administrations of naloxone. These successful responses may lead to a decrease in morbidity and mortality related to the opioid crisis. Future research focused on a well-controlled measurement of the impact of these programs on community health care will be beneficial.

### Limitations

Drug overdose and the resultant morbidity and mortality is multifactorial. This study is limited to evaluation of only one aspect. We cannot quantitatively assess the impact of this program of the total public health crisis.

Our data sources were limited in information. While we are aware of the number of deputies trained per year, the available data does not disclose the retention of trained deputies who are available for naloxone at any given time during the period of the study. However, our purpose was to demonstrate in aggregate, outcomes of the training program which would not be diminished by individuals moving in and out of the affected areas.

The data base provides only a binary response regarding success versus no success. Therefore details that may have provided information on the quality of response or return to baseline were not available.

## Conclusion

We found that the outcome of training non-medical first responders in naloxone administration to be successful with regard to resuscitation of patients with opioid overdose. Extension of this type of training to other non-medical individuals, especially those who are found in close proximity to an environment of substance abuse would be beneficial.

## Data Availability

Data available upon request.

## Abbreviations

CDC: Center for Disease Control
EMS: Emergency Medical Services
CPR: Cardiopulmonary Resuscitation
IRB: Internal Review Board
